# Crystal structure of bis­(μ_2_-meth­anolato-κ*O*:κ*O*)hexa­methyl­bis­(μ_2_-tri­phenyl­acetato-κ*O*:κ*O*′)bis­(μ_2_-tri­phenyl­acetato-κ^2^ *O*,*O*′:κ*O*)dialuminiumdi­lanthanum toluene tetra­solvate

**DOI:** 10.1107/S2056989018015876

**Published:** 2018-11-13

**Authors:** Alexander A. Vinogradov, Dmitrii M. Roitershtein, Mikhail E. Minyaev, Konstantin A. Lyssenko, Ilya E. Nifant’ev

**Affiliations:** a A.V. Topchiev Institute of Petrochemical Synthesis, Russian Academy of Sciences, 29 Leninsky Prospect, 119991, Moscow, Russian Federation; b N.D. Zelinsky Institute of Organic Chemistry, Russian Academy of Sciences, 47 Leninsky Prospect, Moscow, 119991, Russian Federation; c A.N. Nesmeyanov Institute of Organoelement Compounds, Russian Academy of Sciences, 28 Vavilova Str., 119991, Moscow, Russian Federation; d Chemistry Department, M.V. Lomonosov Moscow State, University, 1 Leninskie Gory Str., Building 3, Moscow 119991, Russian Federation

**Keywords:** lanthanum, aluminium, tri­phenyl­acetate, π-complex, coordination compound, crystal structure

## Abstract

The title compound, [Al_2_La_2_(C_20_H_15_O_2_)_4_(CH_3_)_6_(CH_3_O)_2_]·4CH_3_C_6_H_5_ or [{La(Ph_3_CCOO)_2_(Me_3_AlOMe)}_2_]·4CH_3_C_6_H_5_, was formed in a reaction between lanthanum tris­(tetra­methyl­aluminate) and tri­phenyl­acetic acid (1:1) with unintended partial oxidation. The tri­phenyl­acetate ligand exhibits μ_2_-κ^1^ *O*:κ^1^ *O*′ bridging and μ_2_-κ^2^ *O*,*O*′:κ^1^ *O* semi-bridging coordination modes, forming a dimeric La_2_(μ-OCO)_4_ core. The semi-bridging tri­phenyl­acetate group provides additional bonding with an La^3+^ cation *via* the π-system of one of its phenyl rings. The tri­methyl­meth­oxy­aluminate anion, which is coordinated to the La^3+^ cation by its O atom, displays a rather long La—C_Me_ bond. Two toluene mol­ecules are each disordered over two orientations about centres of symmetry with site occupancy factors of 0.5. The title compound represents the first example of an Ln^III^ complex containing both alkyl alkoxide aluminate and π-bounded arene fragments.

## Abstract 2

The complex [{La(Ph_3_CCOO)_2_(Me_3_AlOMe)}_2_] has an La_2_(μ-OCO)_4_ core, contains the carboxyl­ate ligands in μ_2_-κ^1^ *O*:κ^1^ *O*′ bridging and μ_2_-κ^2^ *O*,*O*′:κ^1^ *O* semi-bridging coordination modes, and displays La—C inter­actions with the π-system of a phenyl ring.

## Chemical context   

Heteroleptic tetra­alkyl­aluminate complexes of rare-earth metals attract significant attention because of their intriguing role in the stereospecific polymerization of conjugated dienes (Anwander, 2002 ▸). Stereoregular elastomers obtained in the polymerization process of isoprene and butadiene are fundamentally important for the production of modern wear-resistant rubbers (Friebe *et al.*, 2006 ▸). It is assumed that this type of complex plays the key role in the formation of catalytically active species. Meanwhile, little is known about the structure of such complexes (Fischbach *et al.*, 2006*a* ▸, and reference therein). The exceptionally high oxidative instability of aluminate complexes is one of the reasons for the lack of information on the structures of catalytically active heteroleptic bimetallic Ln–Al complexes.

**Figure 1:**
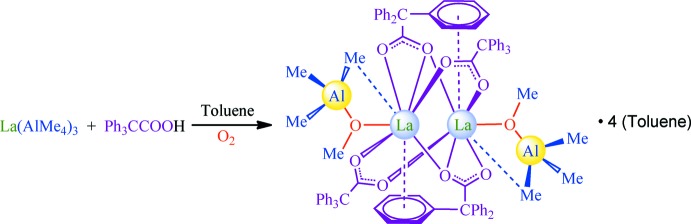
Synthesis of [{La(Ph_3_CCOO)_2_Me_3_AlOMe}_2_]·4(CH_3_C_6_H_5_).

This report describes the product of unintentional oxidation of a carboxyl­ate–aluminate La complex while reacting lanthanum tris­(tetra­methyl­aluminiumate) with the corres­ponding acid (Fig. 1 ▸). This reaction should have led initially to the heteroleptic tri­phenyl­acetate–tetra­methyl­aluminate complex that is supposed to be a model of the active species in the catalyst system. The accidental partial oxidation resulted in the formation of the tri­phenyl­acetate-tri­methyl­meth­oxy­aluminate lanthanum complex [{La(Ph_3_CCOO)_2_Me_3_AlOMe}_2_].
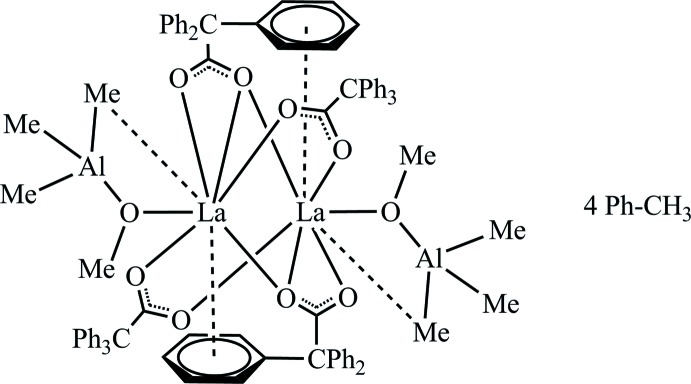


## Structural commentary   

**Figure 2:**
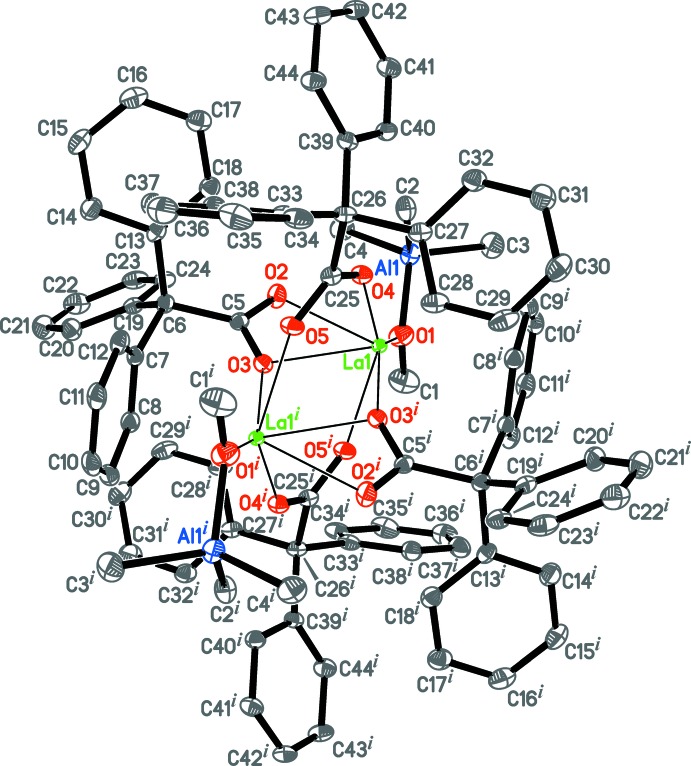
The mol­ecular structure of the {La(Ph_3_CCOO)_2_(Me_3_AlOMe)}_2_ unit in the title compound with displacement ellipsoids drawn at the 30% probability level. Hydrogen atoms and toluene solvent mol­ecules are omitted for clarity. The La—O bonds are shown with thinner solid lines. The La—C inter­actions are not shown. Symmetry code: (i) −*x*, −*y* + 1, −*z* + 1.

**Figure 3:**
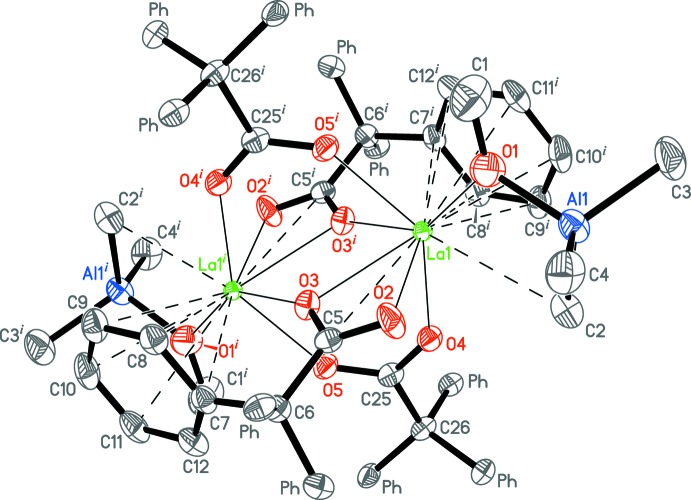
Metal–ligand inter­actions within the {La(Ph_3_CCOO)_2_(Me_3_AlOMe)}_2_ unit. Displacement ellipsoids are drawn at the 50% probability level. Hydrogen atoms are omitted, only C_*ipso*_ atoms (labeled as Ph) are shown for non-coordinating phenyl groups for clarity. The Ln—C contacts are shown with thin dashed lines. Symmetry code: (i) −*x*, −*y* + 1, −*z* + 1.

The asymmetric unit of the title compound consists of half of the dimeric complex [{La(Ph_3_CCOO)_2_(Me_3_AlOMe)}_2_] (Fig. 2 ▸) located on an inversion centre, and three non-coordinating toluene mol­ecules (not shown). Two of the toluene mol­ecules are disordered over inversion centres, having 50% atomic site occupancies. The coordination polyhedron for the La^3+^ cation and its coordination number are rather difficult to determine. Two tri­phenyl­acetate ligands exhibit the μ_2_-κ^1^ *O*:κ^1^ *O*′ bridging coordination mode, but two other ligands display the μ_2_-κ^2^ *O*,*O*′:κ^1^ *O*′ semi-bridging type (Figs. 2 ▸ and 3 ▸; Table 1 ▸). The complex has an La_2_(μ-OCO)_4_ core with an La1⋯La1^i^ distance of 4.0432 (4) Å [symmetry code: (i) −*x*, −*y* + 1, −*z* + 1). Unlike the bridging ligands, the semi-bridging tri­phenyl­acetates demonstrate additional La⋯C contacts with the carb­oxy­lic system (La1⋯C5, La1^i^⋯C5^i^; Fig. 3 ▸; Table 1 ▸). The La^3+^ cation is also coordinated by the π-system of a phenyl ring of the semi-bridging carboxyl­ate ligand (Fig. 3 ▸, atoms C7^i^–C12^i^; Table 1 ▸). The inter­action with the phenyl (Ph) group is close to symmetrical: the La⋯Ph_centroid_ distance is 2.938 (2) Å, the normal to the Ph-ring plane is 2.9353 (16) Å, and the La⋯C_Ph_ bond lengths lie in the range 3.201 (4) to 3.318 (4) Å. Ten crystal structures exhibiting the inter­action of La^3+^ with the π-system of an uncharged C_6_ aromatic ring have been found in the Cambridge Structural Database (CSD, Version 5.39, February 2018 update; Groom *et al.*, 2016 ▸). The corresponding distances in these compounds vary from 2.93 to 3.27 Å for La⋯C_Ar­yl_ and from 2.61 to 2.87 Å for La⋯Ar­yl_centroid_. The La⋯Ph_centroid_ and La⋯C_Ph_ distances in the title compound are therefore the longest, which is likely caused by steric hindrance induced by the presence of many phenyl groups within the inner coordination sphere.

The tri­methyl­metoxyaluminate anions are coordinated to the La^3+^ cations *via* oxygen atoms (La1—O1, La1^i^—O1^i^), and exhibit a slightly distorted tetra­hedral environment about the Al atoms, with an O1—Al1—C2 angle of 100.03 (17)° and with other O—Al—C and C—Al—C bond angles ranging from 108.32 (18) to 113.2 (2)°. The small value for the O1—Al1—C2 angle is due to the additional coordination of the [Al(CH_3_)_3_(OCH_3_)] anion with La^3+^ by the C2 atom (Fig. 3 ▸). However, the La1—C2 bond length [3.042 (4) Å] is rather long compared to those of previously characterized compounds possessing the La–[(μ-Me)_2_AlMe_2_] fragment, which have La—C_Me_ distances lying in the range 2.66 to 2.98 Å with the average value of 2.76 Å (32 compounds with 128 crystallographically independent La—C_Me-Al_ distances retrieved from the CSD). The La1⋯Al1 distance [3.4481 (12) Å] is near to the upper boundary of the La—Al distance range in the aforementioned compounds (from 2.99 to 3.45 Å, with an average of 3.25 Å).

There is only one related compound having the La-[(Alk­yl/Ar­yl)_3_Al(OAlk­yl/OAr­yl)] motif (CSD refcode MIMPED; Giesbrecht *et al.*, 2002 ▸) – {La(O-2,6-^i^Pr_2_C_6_H_3_)[AlMe_2_(μ-Me)(μ-O-2,6-^i^Pr_2_C_6_H_3_)]_2_}. The Al—O [1.864 (3), 1.848 (3) Å], La—O [2.387 (3), 2.367 (3) Å] and Al—C [2.040 (5), 2.053 (6) Å] bond lengths within the LaAl_2_(μ-Me)_2_(μ-OAr­yl)_2_ fragment are similar to those found in the LaAl(μ-Me)(μ-OMe) fragment of the complex reported herein. However, the La1—C2 distance in the title compound (Table 1 ▸) is considerably longer (by 0.24-0.28 Å) than the corresponding La—C distances in MIMPED [2.800 (5), 2.759 (5) Å], presumably due to steric reasons.

In the studied compound, the La—O_Me_ (La1—O1) bond is the shortest, compared to the other La—O bonds, which may be due to delocalization of negative charge on the carb­oxy oxygen atoms and/or steric repulsion of the bulky carboxyl­ate anion.

## Supra­molecular features   

**Figure 4:**
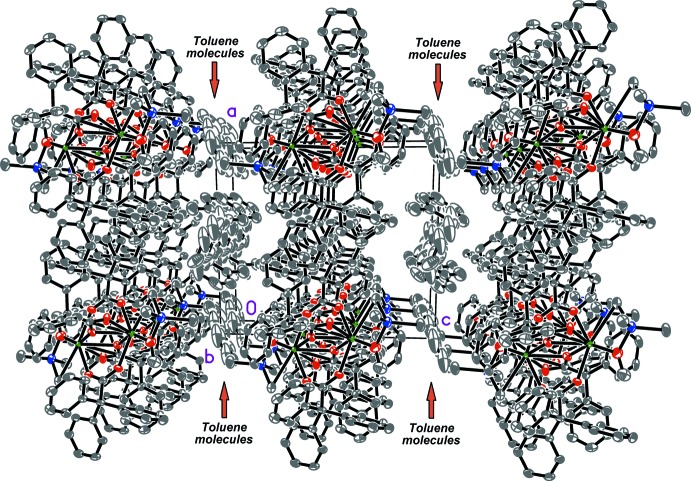
A view along the *b* axis of the crystal packing of the title compound. Displacement ellipsoids are drawn at the 50% probability level. Hydrogen atoms are omitted.

Weak intra- and inter­molecular inter­actions among complex mol­ecules and non-coordinating toluene mol­ecules are mainly represented by the C_Ph_—H··π type (Table 2 ▸). An inter­esting feature of the crystal packing is that the centres of all non-coordinating toluene mol­ecules are located nearly in one plane parallel to the *ab* plane, separating 2D mol­ecular layers of the complex (Fig. 4 ▸).

## Database survey   

The number of crystal structures for rare-earth compounds containing the Ln–C–Al fragment (CSD, Version 5.39, February 2018 update; Groom *et al.*, 2016 ▸) is nearly 250 (upon exclusion of duplicated structures). They are mainly represented by 147 tetra­methyl­aluminates with Ln-[(μ_2_-Me)_2_AlMe_2_] (127 structures), Ln-[(μ_2_-Me)AlMe_2_(μ_2_-Me)]-Ln (11 structures) and Ln-[(μ_2_-Me)AlMe_3_] (9 structures) fragments and by 16 tetra­ethyl­aluminate complexes. This number also includes 18 structures of Ln-[(Alk­yl/Ar­yl)_3_Al(OAlk­yl/OAr­yl) compounds possessing the following structural motifs: [(μ_2_-Me)(μ_2_-OCH_2_^*t*^Bu)AlMe_2_] (AVOYOA, AVOYUG, Occhipinti *et al.*, 2011 ▸; GEQMOF, GEQMUL, Fischbach *et al.*, 2006*b* ▸), [(μ_2_-Me)(μ_2_-O^*t*^Bu)AlMe_2_] (POJNAD, Biagini *et al.*, 1994 ▸; WAPYIV, WAPYOB, Evans *et al.*, 1993*a* ▸; WEHHAS, Evans *et al.*, 1993*b* ▸), [(μ_2_-Me)(μ_2_-O^*i*^Pr)AlMe_2_] (VOLMUF, Liu *et al.*, 2005 ▸), [(μ_2_-Me)(μ_2_-O-2,6-Ph_2_C_6_H_3_)AlMe_2_] (TULCAF, Korobkov & Gambarotta, 2009 ▸), [(μ_2_-Me)(μ_2_-O-2,6-^*i*^Pr_2_C_6_H_3_)AlMe_2_] (LUQZOM, Fischbach *et al.*, 2003 ▸; MIMPED, Giesbrecht *et al.*, 2002 ▸; MOQYOG, Gordon *et al.*, 2002 ▸; PETMUX, Fischbach *et al.*, 2006*c* ▸), [(μ_2_-Et)(μ_2_-O-2,6-^*i*^Pr_2_C_6_H_3_)AlEt_2_] (MIMPIH, Giesbrecht *et al.*, 2002 ▸; ROCHOH, Sommerfeldt *et al.*, 2008 ▸), [(μ_2_-Me)(μ_2_-O-2,6-^*t*^Bu_2_-4-MeC_6_H_2_)AlMe_2_] (ROCGOG, Sommerfeldt *et al.*, 2008 ▸), [(κ^2^ *O*,*O*′-MeOCH_2_CH_2_O)AlMe_3_] (GIZWAN, Evans *et al.*, 1998 ▸). MIMPED is the only La structure among them. A related structure with the {(μ_2_-Me)[μ_2_-κ*O*:κ^2^ *O*,*O*′-(O^*t*^Bu)_3_SiO]AlMe_2_} motif (BEQXUR, Fischbach *et al.*, 2004 ▸) might be also mentioned.

Crystal structures of lanthanide(III) compounds having an η^6^-coordinated uncharged arene system have become numerous over the last two decades, resulting in the description of over 150 crystal structures (see the CSD). Ten structures of such La(III) π-complexes are known: EZIPIM (Giesbrecht *et al.*, 2004 ▸), MALXOM (Deacon *et al.*, 2000 ▸), POKCAU (Gerber *et al.*, 2008 ▸), RILBIZ, RILBUL (Hamidi *et al.*, 2013 ▸), ROMQUG (Filatov *et al.*, 2009 ▸), SOJHAB, SOJHEF, SOJHIJ (Filatov *et al.*, 2008 ▸), ZIDSOV (Butcher *et al.*, 1995 ▸). Crystallographic data for these complexes were used to compare structural parameters of the title compound in the *Structural Commentary* section. Known crystal structures of rare-earth tri­phenyl­acetate complexes are also not numerous, and their number is limited to 16 recent crystal structures: peroxide bis­(tri­phenyl­acetate) complexes QEHBOX, QEHBUD, QEHCEO (Roitershtein *et al.*, 2017 ▸), mono- and binuclear tris­(tri­phenyl­acetate) complexes EPUNIO (Minyaev *et al.*, 2016 ▸), RIKRIO, RIKRUA, RIKSAH, RIKSEL (Roitershtein *et al.*, 2013 ▸), tetra­kis­(tri­phenyl­acetate) complexes and their adducts RIKQUZ, RIKRAG, RIKREK, RIKRIO (Roitershtein *et al.*, 2013 ▸), tri­phenyl­acetate-tetra­ethyl­aluminate compounds RIJVIR, RIJVOX (Roitershtein *et al.*, 2013 ▸) and hepta­nuclear polyligand complexes UVETAR, UVETEV (Sharples *et al.*, 2011 ▸). The tri­phenyl­acetate ligand exhibits terminal κ*O* and κ^2^ *O*,*O*′, bridging μ-κ*O*,κ*O*′, and semi-bridging μ-κ*O*,κ^2^ *O*,*O*′ (the latter is only for the four ate complexes) coordination modes.

Up to date, no complex has been reported that has both an η^6^-coordinated arene ligand and the mixed-ligand alkyl-alkoxide aluminate anion.

## Synthesis and crystallization   

Synthetic operations were carried out under a purified argon atmosphere. Toluene was distilled from sodium/benzo­phenone ketyl, hexane was distilled from Na/K alloy. Tri­phenyl­acetic acid was purified by azeotrope removal of water from its toluene solution with a Dean–Stark trap, followed by crystallization from a cold saturated solution and then by vacuum drying. The complex La(AlMe_4_)_3_ was prepared according to the literature procedure (Zimmermann *et al.*, 2007 ▸).

A solution of Ph_3_CCOOH (0.144 g, 0.50 mmol) in toluene (20 ml) was added to a stirred solution of La(AlMe_4_)_3_ (0.196 g, 0.49 mmol) in toluene (10 ml), producing a suspension, which was stirred overnight at room temperature. The precipitate was removed by deca­ntation and the solution was concentrated to a volume of 10 ml. Slow and careful layering of hexane (40 ml) on the top of the residual solution resulted in the formation of an inseparable compound mixture and a few colourless crystals suitable for X-ray single crystal diffraction analysis.

## Refinement   

Crystal data, data collection and structure refinement details are summarized in Table 3 ▸. The hydrogen atom were positioned geometrically (C—H = 0.95 Å for aromatic, 0.98 Å for methyl H atoms) and refined as riding atoms with *U*_iso_(H) = 1.5*U*_eq_(C) for methyl or 1.2*U*_eq_(C) for aromatic H atoms. A rotating group model was applied for methyl groups. Three reflections (100, 010, 001) were affected by the beam stop, and were therefore omitted from the refinement. Two non-coordinating toluene mol­ecules disordered over inversion centres with occupancy factors of 0.5 were modelled by fitting the phenyl rings to regular hexa­gons, by constraining the C_*ipso*_—C_Me_ bond distances to 1.52 (1) Å, and by using equal anisotropic displacement parameters for atoms C52, C53, C54, C55, C60, C62 and C65.

## Supplementary Material

Crystal structure: contains datablock(s) I. DOI: 10.1107/S2056989018015876/rz5247sup1.cif

Structure factors: contains datablock(s) I. DOI: 10.1107/S2056989018015876/rz5247Isup2.hkl

Supporting information file. DOI: 10.1107/S2056989018015876/rz5247Isup3.cdx

CCDC reference: 1877930

Additional supporting information:  crystallographic information; 3D view; checkCIF report

## Figures and Tables

**Table 1:** Selected bond lengths (Å)

La1—O1	2.336 (3)	La1—C8^i^	3.287 (4)
La1—O2	2.501 (3)	La1—C9^i^	3.246 (4)
La1—O3	2.494 (3)	La1—C10^i^	3.212 (4)
La1—O3^i^	2.403 (2)	La1—C11^i^	3.201 (4)
La1—O4	2.396 (3)	La1—C12^i^	3.239 (4)
La1—O5^i^	2.367 (3)	Al1—O1	1.819 (3)
La1—C2	3.042 (4)	Al1—C2	2.014 (4)
La1—C5	2.892 (4)	Al1—C3	1.990 (5)
La1—C7^i^	3.318 (4)	Al1—C4	1.961 (4)

Symmetry code: (i) 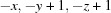.

**Table 2:** Hydrogen-bond geometry (Å, °) *Cg*1, *Cg*2, *Cg*3 and *Cg*4 are the centroids of the C33–C38, C39–C44, C52–C57 and C19–C24 rings, respectively.

*D*—H⋯*A*	*D*—H	H⋯*A*	*D*⋯*A*	*D*—H⋯*A*
C1—H1*C*⋯⋯*Cg*1^i^	0.98	2.69	3.425 (6)	132
C17—H17⋯⋯*Cg*2	0.95	2.71	3.485 (4)	139
C21—H21⋯⋯*Cg*3^ii^	0.95	2.93	3.677 (8)	136
C29—H29⋯⋯*Cg*4	0.95	2.62	3.415 (4)	142
C32—H32⋯⋯*Cg*2	0.95	2.95	3.654 (5)	132
C44—H44⋯⋯*Cg*1	0.95	2.88	3.592 (5)	132

Symmetry codes: (i) 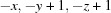; (ii) 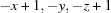.

**Table 3:** Experimental details

Crystal data	
Chemical formula	[Al_2_La_2_(CH_3_)_6_(C_20_H_15_O_2_)_4_(CH_3_O)_2_]·4C_7_H_8_
*M*_r_	2001.86
Crystal system, space group	Triclinic, *P* 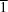
Temperature (K)	100
*a*, *b*, *c* (Å)	13.8404 (6), 14.2089 (6), 14.6084 (7)
α, β, γ (°)	73.198 (1), 81.968 (1), 63.523 (1)
*V* (Å^3^)	2461.54 (19)
*Z*	1
Radiation type	Mo *K*α
μ (mm^−1^)	0.93
Crystal size (mm)	0.43 × 0.17 × 0.14

Data collection	
Diffractometer	Bruker APEXII CCD
Absorption correction	Multi-scan (*SADABS*; Bruker, 2008 ▸)
*T*_min_, *T*_max_	0.713, 0.848
No. of measured, independent and observed [*I* > 2σ(*I*)] reflections	30779, 13082, 10174
*R*_int_	0.065
(sin θ/λ)_max_ (Å^−1^)	0.682

Refinement	
*R*[*F* ^2^ > 2σ(*F* ^2^)], *wR*(*F* ^2^), *S*	0.049, 0.111, 1.01
No. of reflections	13082
No. of parameters	596
No. of restraints	2
H-atom treatment	H-atom parameters constrained
Δρ_max_, Δρ_min_ (e Å^−3^)	1.25, −1.36

Computer programs: *APEX2* (Bruker, 2008 ▸), *SAINT* (Bruker, 2008 ▸), *SHELXS97* (Sheldrick, 2008 ▸), *SHELXL2017* (Sheldrick, 2015 ▸), *SHELXTL* (Sheldrick, 2008 ▸), *SHELXTL* (Sheldrick, 2015 ▸) and *publCIF* (Westrip, 2010 ▸).

## supplementary crystallographic information

### Crystal data

**Table:**

[Al_2_La_2_(CH_3_)_6_(C_20_H_15_O_2_)_4_(CH_3_O)_2_]·4C_7_H_8_	*Z* = 1
*M**_r_* = 2001.86	*F*(000) = 1032
Triclinic, *P*1	*D*_x_ = 1.350 Mg m^−^^3^
*a* = 13.8404 (6) Å	Mo *K*α radiation, λ = 0.71073 Å
*b* = 14.2089 (6) Å	Cell parameters from 4587 reflections
*c* = 14.6084 (7) Å	θ = 2.5–27.3°
α = 73.198 (1)°	µ = 0.93 mm^−^^1^
β = 81.968 (1)°	*T* = 100 K
γ = 63.523 (1)°	Block, colorless
*V* = 2461.54 (19) Å^3^	0.43 × 0.17 × 0.14 mm

### Data collection

**Table:**

Bruker APEXII CCD diffractometer	13082 independent reflections
Radiation source: fine-focus sealed tube	10174 reflections with *I* > 2σ(*I*)
Graphite monochromator	*R*_int_ = 0.065
ω scans	θ_max_ = 29.0°, θ_min_ = 1.9°
Absorption correction: multi-scan (SADABS; Bruker, 2008)	*h* = −18→18
*T*_min_ = 0.713, *T*_max_ = 0.848	*k* = −19→19
30779 measured reflections	*l* = −19→19

### Refinement

**Table:**

Refinement on *F*^2^	Primary atom site location: structure-invariant direct methods
Least-squares matrix: full	Secondary atom site location: difference Fourier map
*R*[*F*^2^ > 2σ(*F*^2^)] = 0.049	Hydrogen site location: inferred from neighbouring sites
*wR*(*F*^2^) = 0.111	H-atom parameters constrained
*S* = 1.01	*w* = 1/[σ^2^(*F*_o_^2^) + (0.0491*P*)^2^] where *P* = (*F*_o_^2^ + 2*F*_c_^2^)/3
13082 reflections	(Δ/σ)_max_ = 0.001
596 parameters	Δρ_max_ = 1.25 e Å^−^^3^
2 restraints	Δρ_min_ = −1.36 e Å^−^^3^

### Special details

**Table:**

Experimental. moisture and air sensitive
Geometry. All esds (except the esd in the dihedral angle between two l.s. planes) are estimated using the full covariance matrix. The cell esds are taken into account individually in the estimation of esds in distances, angles and torsion angles; correlations between esds in cell parameters are only used when they are defined by crystal symmetry. An approximate (isotropic) treatment of cell esds is used for estimating esds involving l.s. planes.
Refinement. Refinement of F^2^ against ALL reflections. The weighted R-factor wR and goodness of fit S are based on F^2^, conventional R-factors R are based on F, with F set to zero for negative F^2^. The threshold expression of F^2^ > 2sigma(F^2^) is used only for calculating R-factors(gt) etc. and is not relevant to the choice of reflections for refinement. R-factors based on F^2^ are statistically about twice as large as those based on F, and R- factors based on ALL data will be even larger.

### Fractional atomic coordinates and isotropic or equivalent isotropic displacement parameters (Å^2^)

**Table:**

	*x*	*y*	*z*	*U*_iso_*/*U*_eq_	Occ. (<1)
La1	−0.03227 (2)	0.49487 (2)	0.37122 (2)	0.01306 (6)	
Al1	−0.12529 (11)	0.40803 (10)	0.21948 (9)	0.0286 (3)	
O1	−0.0005 (2)	0.3834 (3)	0.2697 (2)	0.0386 (7)	
C1	0.0953 (4)	0.3005 (4)	0.2479 (4)	0.0524 (14)	
H1A	0.088611	0.290505	0.185791	0.079*	
H1B	0.109110	0.232657	0.297403	0.079*	
H1C	0.155252	0.320192	0.245107	0.079*	
C2	−0.2300 (4)	0.5359 (3)	0.2694 (3)	0.0336 (10)	
H2A	−0.300473	0.565883	0.239855	0.050*	
H2B	−0.203033	0.591960	0.253632	0.050*	
H2C	−0.237368	0.512220	0.338947	0.050*	
C3	−0.1116 (4)	0.4476 (4)	0.0776 (3)	0.0460 (12)	
H3A	−0.097311	0.384953	0.053500	0.069*	
H3B	−0.051864	0.468840	0.059015	0.069*	
H3C	−0.178880	0.508554	0.050371	0.069*	
C4	−0.1619 (4)	0.2840 (4)	0.2695 (3)	0.0406 (11)	
H4A	−0.110910	0.223099	0.243557	0.061*	
H4B	−0.235322	0.304856	0.250611	0.061*	
H4C	−0.157613	0.261936	0.339463	0.061*	
O2	−0.1140 (2)	0.3706 (2)	0.46652 (19)	0.0320 (7)	
O3	−0.0402 (2)	0.4255 (2)	0.54830 (18)	0.0232 (6)	
C5	−0.0957 (3)	0.3754 (3)	0.5459 (3)	0.0214 (8)	
C6	−0.1398 (3)	0.3264 (3)	0.6418 (3)	0.0220 (8)	
C7	−0.0810 (3)	0.3348 (3)	0.7189 (3)	0.0234 (8)	
C8	0.0289 (3)	0.2683 (3)	0.7329 (3)	0.0271 (8)	
H8	0.064082	0.213330	0.699308	0.033*	
C9	0.0885 (4)	0.2798 (3)	0.7944 (3)	0.0307 (9)	
H9	0.163386	0.233326	0.802589	0.037*	
C10	0.0381 (4)	0.3596 (4)	0.8438 (3)	0.0340 (10)	
H10	0.077959	0.367317	0.886831	0.041*	
C11	−0.0703 (4)	0.4277 (4)	0.8303 (3)	0.0325 (10)	
H11	−0.104716	0.482567	0.864064	0.039*	
C12	−0.1300 (3)	0.4167 (3)	0.7672 (3)	0.0279 (9)	
H12	−0.204174	0.465268	0.757361	0.033*	
C13	−0.2633 (3)	0.3918 (3)	0.6482 (3)	0.0233 (8)	
C14	−0.3158 (3)	0.3660 (4)	0.7337 (3)	0.0342 (10)	
H14	−0.275619	0.309338	0.785860	0.041*	
C15	−0.4279 (4)	0.4235 (4)	0.7427 (3)	0.0391 (11)	
H15	−0.463027	0.406832	0.801646	0.047*	
C16	−0.4875 (4)	0.5041 (4)	0.6668 (4)	0.0387 (11)	
H16	−0.563524	0.542101	0.673235	0.046*	
C17	−0.4364 (3)	0.5292 (3)	0.5818 (3)	0.0340 (10)	
H17	−0.477381	0.584819	0.529442	0.041*	
C18	−0.3253 (3)	0.4737 (3)	0.5721 (3)	0.0284 (9)	
H18	−0.291035	0.491608	0.513002	0.034*	
C19	−0.1186 (3)	0.2076 (3)	0.6507 (3)	0.0249 (8)	
C20	−0.0899 (4)	0.1297 (3)	0.7379 (3)	0.0328 (10)	
H20	−0.078929	0.148647	0.791600	0.039*	
C21	−0.0772 (4)	0.0247 (4)	0.7475 (4)	0.0424 (11)	
H21	−0.059311	−0.026807	0.807948	0.051*	
C22	−0.0904 (4)	−0.0056 (4)	0.6695 (4)	0.0415 (12)	
H22	−0.078727	−0.078289	0.675550	0.050*	
C23	−0.1208 (3)	0.0713 (3)	0.5833 (3)	0.0340 (10)	
H23	−0.131851	0.051784	0.530019	0.041*	
C24	−0.1355 (3)	0.1779 (3)	0.5732 (3)	0.0276 (9)	
H24	−0.157055	0.230159	0.513513	0.033*	
O4	−0.1812 (2)	0.6313 (2)	0.43281 (18)	0.0240 (6)	
O5	−0.1427 (2)	0.6352 (2)	0.57488 (19)	0.0263 (6)	
C25	−0.2039 (3)	0.6717 (3)	0.5035 (3)	0.0227 (8)	
C26	−0.3096 (3)	0.7777 (3)	0.5032 (3)	0.0227 (8)	
C27	−0.2810 (3)	0.8697 (3)	0.4371 (3)	0.0231 (8)	
C28	−0.1853 (3)	0.8743 (3)	0.4508 (3)	0.0301 (9)	
H28	−0.137299	0.820458	0.499793	0.036*	
C29	−0.1600 (3)	0.9568 (3)	0.3933 (3)	0.0337 (10)	
H29	−0.093985	0.957802	0.402696	0.040*	
C30	−0.2283 (4)	1.0370 (4)	0.3232 (3)	0.0368 (10)	
H30	−0.210354	1.093329	0.284112	0.044*	
C31	−0.3234 (4)	1.0339 (4)	0.3106 (3)	0.0382 (11)	
H31	−0.371889	1.089322	0.262629	0.046*	
C32	−0.3499 (3)	0.9516 (3)	0.3667 (3)	0.0304 (9)	
H32	−0.416150	0.951342	0.356674	0.037*	
C33	−0.3352 (3)	0.7907 (3)	0.6049 (3)	0.0257 (8)	
C34	−0.3485 (3)	0.8825 (3)	0.6307 (3)	0.0314 (9)	
H34	−0.339816	0.940681	0.584050	0.038*	
C35	−0.3746 (4)	0.8894 (4)	0.7256 (4)	0.0437 (12)	
H35	−0.383374	0.952358	0.742752	0.052*	
C36	−0.3876 (4)	0.8057 (4)	0.7943 (3)	0.0447 (12)	
H36	−0.404321	0.810532	0.858570	0.054*	
C37	−0.3762 (3)	0.7144 (4)	0.7688 (3)	0.0382 (11)	
H37	−0.386189	0.656965	0.815430	0.046*	
C38	−0.3502 (3)	0.7074 (4)	0.6750 (3)	0.0311 (9)	
H38	−0.342435	0.644566	0.658109	0.037*	
C39	−0.4077 (3)	0.7783 (3)	0.4627 (3)	0.0229 (8)	
C40	−0.4009 (3)	0.7506 (3)	0.3772 (3)	0.0257 (8)	
H40	−0.334014	0.729044	0.343230	0.031*	
C41	−0.4902 (3)	0.7541 (3)	0.3408 (3)	0.0300 (9)	
H41	−0.482883	0.732093	0.283689	0.036*	
C42	−0.5896 (3)	0.7893 (3)	0.3865 (3)	0.0316 (9)	
H42	−0.650696	0.792166	0.361079	0.038*	
C43	−0.5981 (3)	0.8199 (4)	0.4696 (3)	0.0334 (10)	
H43	−0.666067	0.844944	0.501464	0.040*	
C44	−0.5089 (3)	0.8148 (3)	0.5072 (3)	0.0287 (9)	
H44	−0.516858	0.836590	0.564489	0.034*	
C45	0.3342 (4)	−0.0048 (5)	1.0374 (4)	0.0480 (13)	
C46	0.3902 (4)	−0.0087 (5)	0.9511 (4)	0.0585 (15)	
H46	0.427274	−0.076125	0.934371	0.070*	
C47	0.3928 (5)	0.0844 (6)	0.8891 (5)	0.074 (2)	
H47	0.430937	0.080655	0.829926	0.088*	
C48	0.3409 (6)	0.1817 (6)	0.9125 (5)	0.075 (2)	
H48	0.344572	0.245235	0.870865	0.090*	
C49	0.2825 (5)	0.1870 (5)	0.9978 (5)	0.0677 (19)	
H49	0.244438	0.254804	1.013625	0.081*	
C50	0.2794 (4)	0.0939 (5)	1.0599 (4)	0.0563 (15)	
H50	0.239503	0.098150	1.118164	0.068*	
C51	0.3334 (5)	−0.1067 (5)	1.1063 (4)	0.0719 (19)	
H51A	0.398951	−0.169935	1.096588	0.108*	
H51B	0.269827	−0.115018	1.094882	0.108*	
H51C	0.330958	−0.101436	1.172030	0.108*	
C52	1.0278 (10)	−0.0489 (11)	−0.0048 (11)	0.153 (5)	0.5
C53	1.0634 (11)	0.0326 (16)	−0.0286 (11)	0.153 (5)	0.5
H53	1.131932	0.020149	−0.058622	0.184*	0.5
C54	0.9986 (15)	0.1324 (13)	−0.0086 (9)	0.153 (5)	0.5
H54	1.022959	0.188084	−0.024851	0.184*	0.5
C55	0.8983 (14)	0.1506 (8)	0.0354 (9)	0.153 (5)	0.5
H55	0.854108	0.218810	0.049064	0.184*	0.5
C56	0.8628 (8)	0.0691 (11)	0.0592 (7)	0.088 (5)	0.5
H56	0.794229	0.081601	0.089209	0.106*	0.5
C57	0.9275 (10)	−0.0306 (9)	0.0391 (8)	0.066 (4)	0.5
H57	0.903200	−0.086335	0.055439	0.079*	0.5
C58	1.1043 (16)	−0.1507 (13)	−0.0328 (18)	0.167 (13)	0.5
H58A	1.147324	−0.132880	−0.088484	0.251*	0.5
H58B	1.063330	−0.184832	−0.048928	0.251*	0.5
H58C	1.152284	−0.201064	0.020591	0.251*	0.5
C59	0.4395 (9)	0.4875 (10)	−0.0082 (9)	0.088 (6)	0.5
C60	0.3781 (7)	0.5983 (10)	−0.0446 (7)	0.153 (5)	0.5
H60	0.308346	0.623626	−0.069200	0.184*	0.5
C61	0.4186 (10)	0.6720 (8)	−0.0449 (8)	0.079 (4)	0.5
H61	0.376605	0.747699	−0.069740	0.095*	0.5
C62	0.5206 (11)	0.6349 (10)	−0.0089 (8)	0.153 (5)	0.5
H62	0.548327	0.685295	−0.009072	0.184*	0.5
C63	0.5821 (8)	0.5242 (11)	0.0275 (8)	0.134 (11)	0.5
H63	0.651790	0.498818	0.052136	0.161*	0.5
C64	0.5415 (9)	0.4504 (8)	0.0278 (8)	0.104 (7)	0.5
H64	0.583532	0.374743	0.052676	0.125*	0.5
C65	0.421 (2)	0.4005 (15)	−0.0297 (12)	0.153 (5)	0.5
H65A	0.409788	0.418014	−0.098456	0.230*	0.5
H65B	0.357617	0.395283	0.005788	0.230*	0.5
H65C	0.484644	0.330811	−0.010675	0.230*	0.5

### Atomic displacement parameters (Å^2^)

**Table:**

	*U*^11^	*U*^22^	*U*^33^	*U*^12^	*U*^13^	*U*^23^
La1	0.01304 (9)	0.01478 (10)	0.01329 (9)	−0.00636 (7)	−0.00039 (6)	−0.00557 (7)
Al1	0.0374 (7)	0.0314 (7)	0.0258 (6)	−0.0203 (6)	0.0014 (5)	−0.0117 (5)
O1	0.0376 (18)	0.049 (2)	0.0400 (18)	−0.0229 (16)	0.0015 (14)	−0.0198 (15)
C1	0.052 (3)	0.047 (3)	0.067 (4)	−0.018 (3)	−0.012 (3)	−0.025 (3)
C2	0.047 (3)	0.027 (2)	0.029 (2)	−0.018 (2)	0.0001 (19)	−0.0075 (18)
C3	0.060 (3)	0.065 (3)	0.030 (2)	−0.038 (3)	−0.004 (2)	−0.016 (2)
C4	0.047 (3)	0.041 (3)	0.047 (3)	−0.028 (2)	−0.005 (2)	−0.014 (2)
O2	0.0475 (18)	0.0466 (18)	0.0183 (14)	−0.0334 (16)	−0.0007 (13)	−0.0097 (13)
O3	0.0256 (14)	0.0203 (13)	0.0257 (14)	−0.0117 (11)	−0.0054 (11)	−0.0034 (11)
C5	0.0194 (18)	0.0199 (18)	0.025 (2)	−0.0081 (15)	0.0007 (15)	−0.0069 (15)
C6	0.0241 (19)	0.0241 (19)	0.0222 (19)	−0.0139 (16)	0.0003 (15)	−0.0067 (15)
C7	0.029 (2)	0.027 (2)	0.0189 (18)	−0.0185 (17)	−0.0003 (15)	−0.0031 (15)
C8	0.032 (2)	0.030 (2)	0.024 (2)	−0.0181 (18)	−0.0006 (16)	−0.0053 (16)
C9	0.037 (2)	0.034 (2)	0.027 (2)	−0.023 (2)	−0.0081 (18)	0.0000 (17)
C10	0.050 (3)	0.042 (3)	0.022 (2)	−0.032 (2)	−0.0093 (19)	−0.0013 (18)
C11	0.049 (3)	0.040 (3)	0.019 (2)	−0.027 (2)	0.0043 (18)	−0.0113 (18)
C12	0.036 (2)	0.031 (2)	0.023 (2)	−0.0203 (19)	0.0058 (17)	−0.0101 (17)
C13	0.026 (2)	0.027 (2)	0.025 (2)	−0.0165 (17)	0.0047 (16)	−0.0115 (16)
C14	0.031 (2)	0.033 (2)	0.035 (2)	−0.0144 (19)	0.0036 (19)	−0.0057 (19)
C15	0.036 (2)	0.043 (3)	0.041 (3)	−0.022 (2)	0.014 (2)	−0.012 (2)
C16	0.027 (2)	0.036 (3)	0.056 (3)	−0.014 (2)	0.003 (2)	−0.016 (2)
C17	0.031 (2)	0.029 (2)	0.044 (3)	−0.0140 (19)	−0.004 (2)	−0.0091 (19)
C18	0.029 (2)	0.032 (2)	0.027 (2)	−0.0155 (18)	0.0020 (17)	−0.0097 (17)
C19	0.0196 (19)	0.027 (2)	0.031 (2)	−0.0126 (16)	0.0006 (16)	−0.0089 (16)
C20	0.039 (2)	0.030 (2)	0.034 (2)	−0.021 (2)	−0.0053 (19)	−0.0018 (18)
C21	0.048 (3)	0.032 (3)	0.050 (3)	−0.024 (2)	−0.008 (2)	0.001 (2)
C22	0.035 (3)	0.024 (2)	0.067 (3)	−0.014 (2)	−0.005 (2)	−0.008 (2)
C23	0.025 (2)	0.030 (2)	0.055 (3)	−0.0130 (18)	0.000 (2)	−0.021 (2)
C24	0.022 (2)	0.026 (2)	0.038 (2)	−0.0117 (17)	−0.0013 (17)	−0.0103 (17)
O4	0.0219 (13)	0.0260 (14)	0.0255 (14)	−0.0085 (11)	−0.0015 (11)	−0.0110 (11)
O5	0.0196 (13)	0.0295 (15)	0.0269 (15)	−0.0044 (11)	−0.0029 (11)	−0.0120 (12)
C25	0.0205 (19)	0.0220 (19)	0.028 (2)	−0.0091 (15)	0.0005 (15)	−0.0101 (16)
C26	0.0171 (18)	0.0212 (19)	0.031 (2)	−0.0064 (15)	−0.0023 (15)	−0.0105 (16)
C27	0.0201 (18)	0.0227 (19)	0.030 (2)	−0.0083 (15)	−0.0005 (15)	−0.0125 (16)
C28	0.022 (2)	0.028 (2)	0.043 (3)	−0.0088 (17)	−0.0016 (18)	−0.0158 (19)
C29	0.024 (2)	0.034 (2)	0.053 (3)	−0.0145 (19)	0.0061 (19)	−0.024 (2)
C30	0.040 (3)	0.031 (2)	0.047 (3)	−0.022 (2)	0.010 (2)	−0.015 (2)
C31	0.040 (3)	0.030 (2)	0.046 (3)	−0.017 (2)	−0.007 (2)	−0.004 (2)
C32	0.028 (2)	0.027 (2)	0.039 (2)	−0.0135 (18)	−0.0037 (18)	−0.0078 (18)
C33	0.0177 (18)	0.033 (2)	0.026 (2)	−0.0042 (16)	−0.0031 (15)	−0.0161 (17)
C34	0.022 (2)	0.033 (2)	0.038 (2)	−0.0033 (17)	−0.0048 (17)	−0.0196 (19)
C35	0.035 (3)	0.046 (3)	0.046 (3)	−0.003 (2)	−0.007 (2)	−0.028 (2)
C36	0.035 (3)	0.065 (3)	0.031 (3)	−0.010 (2)	0.002 (2)	−0.027 (2)
C37	0.032 (2)	0.050 (3)	0.026 (2)	−0.011 (2)	−0.0008 (18)	−0.011 (2)
C38	0.027 (2)	0.038 (2)	0.027 (2)	−0.0113 (19)	0.0001 (17)	−0.0112 (18)
C39	0.0200 (18)	0.0205 (19)	0.027 (2)	−0.0074 (15)	−0.0043 (15)	−0.0049 (15)
C40	0.0205 (19)	0.029 (2)	0.027 (2)	−0.0090 (16)	−0.0006 (16)	−0.0084 (17)
C41	0.028 (2)	0.033 (2)	0.030 (2)	−0.0114 (18)	−0.0058 (17)	−0.0111 (18)
C42	0.024 (2)	0.038 (2)	0.038 (2)	−0.0156 (19)	−0.0055 (18)	−0.0116 (19)
C43	0.019 (2)	0.038 (2)	0.044 (3)	−0.0092 (18)	0.0008 (18)	−0.017 (2)
C44	0.023 (2)	0.030 (2)	0.032 (2)	−0.0074 (17)	−0.0003 (17)	−0.0139 (18)
C45	0.035 (3)	0.063 (4)	0.046 (3)	−0.023 (3)	−0.008 (2)	−0.006 (3)
C46	0.043 (3)	0.071 (4)	0.052 (3)	−0.019 (3)	0.004 (3)	−0.012 (3)
C47	0.058 (4)	0.099 (6)	0.054 (4)	−0.043 (4)	−0.009 (3)	0.014 (4)
C48	0.081 (5)	0.069 (5)	0.081 (5)	−0.047 (4)	−0.048 (4)	0.019 (4)
C49	0.069 (4)	0.054 (4)	0.081 (5)	−0.015 (3)	−0.043 (4)	−0.018 (3)
C50	0.048 (3)	0.074 (4)	0.050 (3)	−0.021 (3)	−0.014 (3)	−0.022 (3)
C51	0.071 (4)	0.081 (5)	0.064 (4)	−0.046 (4)	−0.012 (3)	0.010 (3)
C52	0.237 (14)	0.187 (12)	0.044 (5)	−0.111 (12)	−0.030 (6)	0.006 (6)
C53	0.237 (14)	0.187 (12)	0.044 (5)	−0.111 (12)	−0.030 (6)	0.006 (6)
C54	0.237 (14)	0.187 (12)	0.044 (5)	−0.111 (12)	−0.030 (6)	0.006 (6)
C55	0.237 (14)	0.187 (12)	0.044 (5)	−0.111 (12)	−0.030 (6)	0.006 (6)
C56	0.077 (10)	0.133 (15)	0.049 (8)	−0.037 (11)	−0.010 (7)	−0.023 (10)
C57	0.085 (9)	0.077 (9)	0.061 (8)	−0.075 (8)	−0.048 (7)	0.034 (6)
C58	0.19 (2)	0.063 (12)	0.20 (2)	0.052 (12)	−0.125 (19)	−0.094 (15)
C59	0.090 (12)	0.167 (18)	0.083 (12)	−0.115 (14)	0.040 (9)	−0.058 (12)
C60	0.237 (14)	0.187 (12)	0.044 (5)	−0.111 (12)	−0.030 (6)	0.006 (6)
C61	0.072 (9)	0.066 (9)	0.099 (11)	−0.038 (8)	0.035 (8)	−0.023 (8)
C62	0.237 (14)	0.187 (12)	0.044 (5)	−0.111 (12)	−0.030 (6)	0.006 (6)
C63	0.112 (15)	0.29 (3)	0.127 (16)	−0.16 (2)	0.072 (12)	−0.15 (2)
C64	0.095 (13)	0.22 (3)	0.046 (9)	−0.107 (16)	0.015 (7)	−0.042 (12)
C65	0.237 (14)	0.187 (12)	0.044 (5)	−0.111 (12)	−0.030 (6)	0.006 (6)

### Geometric parameters (Å, º)

**Table:**

La1—O1	2.336 (3)	C27—C32	1.383 (5)
La1—O2	2.501 (3)	C27—C28	1.400 (5)
La1—O3	2.494 (3)	C28—C29	1.383 (6)
La1—O3^i^	2.403 (2)	C28—H28	0.9500
La1—O4	2.396 (3)	C29—C30	1.370 (6)
La1—O5^i^	2.367 (3)	C29—H29	0.9500
La1—C2	3.042 (4)	C30—C31	1.376 (6)
La1—C5	2.892 (4)	C30—H30	0.9500
La1—C7^i^	3.318 (4)	C31—C32	1.385 (6)
La1—C8^i^	3.287 (4)	C31—H31	0.9500
La1—C9^i^	3.246 (4)	C32—H32	0.9500
La1—C10^i^	3.212 (4)	C33—C34	1.388 (5)
La1—C11^i^	3.201 (4)	C33—C38	1.398 (6)
La1—C12^i^	3.239 (4)	C34—C35	1.403 (6)
Al1—O1	1.819 (3)	C34—H34	0.9500
Al1—C2	2.014 (4)	C35—C36	1.380 (7)
Al1—C3	1.990 (5)	C35—H35	0.9500
Al1—C4	1.961 (4)	C36—C37	1.387 (7)
La1—Al1	3.4481 (12)	C36—H36	0.9500
La1—La1^i^	4.0432 (4)	C37—C38	1.388 (6)
O1—C1	1.398 (6)	C37—H37	0.9500
C1—H1A	0.9800	C38—H38	0.9500
C1—H1B	0.9800	C39—C40	1.394 (5)
C1—H1C	0.9800	C39—C44	1.396 (5)
C2—H2A	0.9800	C40—C41	1.388 (5)
C2—H2B	0.9800	C40—H40	0.9500
C2—H2C	0.9800	C41—C42	1.382 (6)
C3—H3A	0.9800	C41—H41	0.9500
C3—H3B	0.9800	C42—C43	1.378 (6)
C3—H3C	0.9800	C42—H42	0.9500
C4—H4A	0.9800	C43—C44	1.385 (6)
C4—H4B	0.9800	C43—H43	0.9500
C4—H4C	0.9800	C44—H44	0.9500
O2—C5	1.247 (4)	C45—C50	1.378 (8)
O3—C5	1.269 (4)	C45—C46	1.387 (7)
C5—C6	1.548 (5)	C45—C51	1.509 (7)
C6—C7	1.538 (5)	C46—C47	1.383 (8)
C6—C13	1.544 (5)	C46—H46	0.9500
C6—C19	1.548 (5)	C47—C48	1.365 (9)
C7—C8	1.394 (5)	C47—H47	0.9500
C7—C12	1.397 (5)	C48—C49	1.389 (9)
C8—C9	1.389 (5)	C48—H48	0.9500
C8—H8	0.9500	C49—C50	1.385 (9)
C9—C10	1.385 (6)	C49—H49	0.9500
C9—H9	0.9500	C50—H50	0.9500
C10—C11	1.379 (6)	C51—H51A	0.9800
C10—H10	0.9500	C51—H51B	0.9800
C11—C12	1.403 (5)	C51—H51C	0.9800
C11—H11	0.9500	C52—C53	1.3900
C12—H12	0.9500	C52—C57	1.3900
C13—C14	1.394 (5)	C52—C58	1.496 (8)
C13—C18	1.397 (5)	C53—C54	1.3900
C14—C15	1.402 (6)	C53—H53	0.9500
C14—H14	0.9500	C54—C55	1.3900
C15—C16	1.378 (6)	C54—H54	0.9500
C15—H15	0.9500	C55—C56	1.3900
C16—C17	1.376 (6)	C55—H55	0.9500
C16—H16	0.9500	C56—C57	1.3900
C17—C18	1.389 (6)	C56—H56	0.9500
C17—H17	0.9500	C57—H57	0.9500
C18—H18	0.9500	C58—H58A	0.9800
C19—C20	1.393 (6)	C58—H58B	0.9800
C19—C24	1.397 (5)	C58—H58C	0.9800
C20—C21	1.388 (6)	C59—C60	1.3900
C20—H20	0.9500	C59—C64	1.3900
C21—C22	1.389 (7)	C59—C65	1.489 (9)
C21—H21	0.9500	C60—C61	1.3900
C22—C23	1.379 (6)	C60—H60	0.9500
C22—H22	0.9500	C61—C62	1.3900
C23—C24	1.400 (5)	C61—H61	0.9500
C23—H23	0.9500	C62—C63	1.3900
C24—H24	0.9500	C62—H62	0.9500
O4—C25	1.260 (4)	C63—C64	1.3900
O5—C25	1.271 (4)	C63—H63	0.9500
C25—C26	1.563 (5)	C64—H64	0.9500
C26—C33	1.522 (5)	C65—H65A	0.9800
C26—C39	1.550 (5)	C65—H65B	0.9800
C26—C27	1.557 (5)	C65—H65C	0.9800
			
O1—La1—O5^i^	82.41 (10)	C10—C9—H9	120.2
O1—La1—O4	139.15 (10)	C8—C9—H9	120.2
O5^i^—La1—O4	135.39 (9)	C11—C10—C9	119.8 (4)
O1—La1—O3^i^	147.59 (10)	C11—C10—H10	120.1
O5^i^—La1—O3^i^	71.61 (9)	C9—C10—H10	120.1
O4—La1—O3^i^	72.32 (9)	C10—C11—C12	120.6 (4)
O1—La1—O3	121.24 (10)	C10—C11—H11	119.7
O5^i^—La1—O3	71.46 (9)	C12—C11—H11	119.7
O4—La1—O3	71.58 (9)	C7—C12—C11	120.3 (4)
O3^i^—La1—O3	68.70 (10)	C7—C12—H12	119.8
O1—La1—O2	78.88 (10)	C11—C12—H12	119.8
O5^i^—La1—O2	91.47 (10)	C14—C13—C18	118.4 (4)
O4—La1—O2	84.39 (9)	C14—C13—C6	118.1 (4)
O3^i^—La1—O2	119.83 (8)	C18—C13—C6	123.5 (3)
O3—La1—O2	51.29 (8)	C13—C14—C15	120.0 (4)
O1—La1—C5	101.05 (11)	C13—C14—H14	120.0
O5^i^—La1—C5	82.20 (10)	C15—C14—H14	120.0
O4—La1—C5	75.29 (10)	C16—C15—C14	120.6 (4)
O3^i^—La1—C5	94.40 (9)	C16—C15—H15	119.7
O3—La1—C5	25.94 (9)	C14—C15—H15	119.7
O2—La1—C5	25.44 (9)	C17—C16—C15	119.7 (4)
O1—La1—C2	64.73 (11)	C17—C16—H16	120.1
O5^i^—La1—C2	144.59 (10)	C15—C16—H16	120.1
O4—La1—C2	74.60 (10)	C16—C17—C18	120.3 (4)
O3^i^—La1—C2	143.78 (10)	C16—C17—H17	119.9
O3—La1—C2	113.77 (10)	C18—C17—H17	119.9
O2—La1—C2	70.40 (10)	C17—C18—C13	120.9 (4)
C5—La1—C2	91.12 (11)	C17—C18—H18	119.5
O1—La1—C11^i^	67.47 (11)	C13—C18—H18	119.5
O5^i^—La1—C11^i^	89.35 (11)	C20—C19—C24	118.4 (4)
O4—La1—C11^i^	117.69 (10)	C20—C19—C6	120.8 (3)
O3^i^—La1—C11^i^	92.66 (9)	C24—C19—C6	120.7 (4)
O3—La1—C11^i^	156.40 (10)	C21—C20—C19	120.9 (4)
O2—La1—C11^i^	145.92 (9)	C21—C20—H20	119.5
C5—La1—C11^i^	166.69 (10)	C19—C20—H20	119.5
C2—La1—C11^i^	89.83 (11)	C20—C21—C22	120.6 (4)
O1—La1—C10^i^	73.06 (11)	C20—C21—H21	119.7
O5^i^—La1—C10^i^	114.19 (11)	C22—C21—H21	119.7
O4—La1—C10^i^	97.09 (11)	C23—C22—C21	119.0 (4)
O3^i^—La1—C10^i^	99.91 (9)	C23—C22—H22	120.5
O3—La1—C10^i^	165.67 (10)	C21—C22—H22	120.5
O2—La1—C10^i^	138.28 (9)	C22—C23—C24	120.9 (4)
C5—La1—C10^i^	160.95 (11)	C22—C23—H23	119.6
C2—La1—C10^i^	69.92 (11)	C24—C23—H23	119.6
C11^i^—La1—C10^i^	24.84 (11)	C19—C24—C23	120.2 (4)
O1—La1—C12^i^	86.54 (10)	C19—C24—H24	119.9
O5^i^—La1—C12^i^	75.20 (10)	C23—C24—H24	119.9
O4—La1—C12^i^	114.12 (10)	C25—O4—La1	139.1 (2)
O3^i^—La1—C12^i^	68.61 (9)	C25—O5—La1^i^	141.2 (2)
O3—La1—C12^i^	132.01 (9)	O4—C25—O5	124.1 (3)
O2—La1—C12^i^	161.48 (10)	O4—C25—C26	119.6 (3)
C5—La1—C12^i^	155.02 (11)	O5—C25—C26	116.2 (3)
C2—La1—C12^i^	113.49 (11)	C33—C26—C39	109.6 (3)
C11^i^—La1—C12^i^	25.16 (10)	C33—C26—C27	111.5 (3)
C10^i^—La1—C12^i^	44.00 (11)	C39—C26—C27	109.9 (3)
O1—La1—C9^i^	96.80 (11)	C33—C26—C25	109.0 (3)
O5^i^—La1—C9^i^	125.80 (10)	C39—C26—C25	113.0 (3)
O4—La1—C9^i^	74.39 (10)	C27—C26—C25	103.8 (3)
O3^i^—La1—C9^i^	83.65 (9)	C32—C27—C28	117.8 (4)
O3—La1—C9^i^	141.16 (9)	C32—C27—C26	122.1 (3)
O2—La1—C9^i^	141.89 (10)	C28—C27—C26	120.0 (3)
C5—La1—C9^i^	148.71 (11)	C29—C28—C27	120.4 (4)
C2—La1—C9^i^	73.57 (10)	C29—C28—H28	119.8
C11^i^—La1—C9^i^	43.54 (11)	C27—C28—H28	119.8
C10^i^—La1—C9^i^	24.77 (11)	C30—C29—C28	121.3 (4)
C12^i^—La1—C9^i^	50.79 (11)	C30—C29—H29	119.3
O1—La1—C8^i^	115.26 (11)	C28—C29—H29	119.3
O5^i^—La1—C8^i^	109.86 (10)	C29—C30—C31	118.4 (4)
O4—La1—C8^i^	71.71 (10)	C29—C30—H30	120.8
O3^i^—La1—C8^i^	59.29 (9)	C31—C30—H30	120.8
O3—La1—C8^i^	122.88 (9)	C30—C31—C32	121.2 (4)
O2—La1—C8^i^	155.27 (10)	C30—C31—H31	119.4
C5—La1—C8^i^	142.66 (10)	C32—C31—H31	119.4
C2—La1—C8^i^	96.47 (10)	C27—C32—C31	120.8 (4)
C11^i^—La1—C8^i^	50.24 (11)	C27—C32—H32	119.6
C10^i^—La1—C8^i^	43.27 (10)	C31—C32—H32	119.6
C12^i^—La1—C8^i^	42.94 (10)	C34—C33—C38	118.4 (4)
C9^i^—La1—C8^i^	24.55 (9)	C34—C33—C26	123.6 (4)
O1—La1—C7^i^	110.09 (10)	C38—C33—C26	118.0 (3)
O5^i^—La1—C7^i^	85.71 (10)	C33—C34—C35	120.1 (4)
O4—La1—C7^i^	90.93 (9)	C33—C34—H34	119.9
O3^i^—La1—C7^i^	50.27 (9)	C35—C34—H34	119.9
O3—La1—C7^i^	118.89 (8)	C36—C35—C34	120.7 (4)
O2—La1—C7^i^	170.08 (9)	C36—C35—H35	119.6
C5—La1—C7^i^	144.67 (10)	C34—C35—H35	119.6
C2—La1—C7^i^	116.78 (10)	C35—C36—C37	119.5 (4)
C11^i^—La1—C7^i^	43.70 (10)	C35—C36—H36	120.2
C10^i^—La1—C7^i^	50.94 (10)	C37—C36—H36	120.2
C12^i^—La1—C7^i^	24.56 (9)	C36—C37—C38	119.8 (5)
C9^i^—La1—C7^i^	43.50 (10)	C36—C37—H37	120.1
C8^i^—La1—C7^i^	24.35 (9)	C38—C37—H37	120.1
O1—La1—Al1	29.38 (8)	C37—C38—C33	121.3 (4)
O5^i^—La1—Al1	110.37 (7)	C37—C38—H38	119.3
O4—La1—Al1	109.78 (6)	C33—C38—H38	119.3
O3^i^—La1—Al1	169.94 (6)	C40—C39—C44	117.2 (3)
O3—La1—Al1	121.36 (6)	C40—C39—C26	121.8 (3)
O2—La1—Al1	70.20 (6)	C44—C39—C26	120.8 (3)
C5—La1—Al1	95.64 (8)	C41—C40—C39	121.1 (4)
C2—La1—Al1	35.46 (8)	C41—C40—H40	119.5
C11^i^—La1—Al1	77.62 (7)	C39—C40—H40	119.5
C10^i^—La1—Al1	70.17 (7)	C42—C41—C40	120.9 (4)
C12^i^—La1—Al1	102.03 (7)	C42—C41—H41	119.6
C9^i^—La1—Al1	87.46 (7)	C40—C41—H41	119.6
C8^i^—La1—Al1	111.50 (7)	C43—C42—C41	118.6 (4)
C7^i^—La1—Al1	119.68 (7)	C43—C42—H42	120.7
O1—La1—La1^i^	145.25 (8)	C41—C42—H42	120.7
O5^i^—La1—La1^i^	67.44 (6)	C42—C43—C44	120.8 (4)
O4—La1—La1^i^	67.95 (6)	C42—C43—H43	119.6
O3^i^—La1—La1^i^	35.07 (6)	C44—C43—H43	119.6
O3—La1—La1^i^	33.63 (6)	C43—C44—C39	121.4 (4)
O2—La1—La1^i^	84.84 (6)	C43—C44—H44	119.3
C5—La1—La1^i^	59.40 (7)	C39—C44—H44	119.3
C2—La1—La1^i^	136.72 (8)	C50—C45—C46	118.9 (6)
C11^i^—La1—La1^i^	126.35 (7)	C50—C45—C51	120.2 (6)
C10^i^—La1—La1^i^	134.41 (7)	C46—C45—C51	120.9 (6)
C12^i^—La1—La1^i^	101.30 (7)	C47—C46—C45	120.8 (6)
C9^i^—La1—La1^i^	114.49 (7)	C47—C46—H46	119.6
C8^i^—La1—La1^i^	91.79 (7)	C45—C46—H46	119.6
C7^i^—La1—La1^i^	85.30 (6)	C48—C47—C46	120.3 (7)
Al1—La1—La1^i^	154.99 (2)	C48—C47—H47	119.8
O1—Al1—C4	111.77 (18)	C46—C47—H47	119.8
O1—Al1—C3	108.32 (18)	C47—C48—C49	119.4 (6)
C4—Al1—C3	113.2 (2)	C47—C48—H48	120.3
O1—Al1—C2	100.03 (17)	C49—C48—H48	120.3
C4—Al1—C2	110.7 (2)	C50—C49—C48	120.4 (6)
C3—Al1—C2	112.0 (2)	C50—C49—H49	119.8
O1—Al1—La1	39.05 (10)	C48—C49—H49	119.8
C4—Al1—La1	120.09 (15)	C45—C50—C49	120.2 (6)
C3—Al1—La1	124.87 (14)	C45—C50—H50	119.9
C2—Al1—La1	61.19 (13)	C49—C50—H50	119.9
C1—O1—Al1	118.0 (3)	C45—C51—H51A	109.5
C1—O1—La1	130.3 (3)	C45—C51—H51B	109.5
Al1—O1—La1	111.57 (15)	H51A—C51—H51B	109.5
O1—C1—H1A	109.5	C45—C51—H51C	109.5
O1—C1—H1B	109.5	H51A—C51—H51C	109.5
H1A—C1—H1B	109.5	H51B—C51—H51C	109.5
O1—C1—H1C	109.5	C53—C52—C57	120.0
H1A—C1—H1C	109.5	C53—C52—C58	114.2 (15)
H1B—C1—H1C	109.5	C57—C52—C58	125.7 (15)
Al1—C2—La1	83.34 (15)	C54—C53—C52	120.0
Al1—C2—H2A	109.5	C54—C53—H53	120.0
La1—C2—H2A	166.5	C52—C53—H53	120.0
Al1—C2—H2B	109.5	C55—C54—C53	120.0
La1—C2—H2B	60.7	C55—C54—H54	120.0
H2A—C2—H2B	109.5	C53—C54—H54	120.0
Al1—C2—H2C	109.5	C54—C55—C56	120.0
La1—C2—H2C	68.5	C54—C55—H55	120.0
H2A—C2—H2C	109.5	C56—C55—H55	120.0
H2B—C2—H2C	109.5	C55—C56—C57	120.0
Al1—C3—H3A	109.5	C55—C56—H56	120.0
Al1—C3—H3B	109.5	C57—C56—H56	120.0
H3A—C3—H3B	109.5	C56—C57—C52	120.0
Al1—C3—H3C	109.5	C56—C57—H57	120.0
H3A—C3—H3C	109.5	C52—C57—H57	120.0
H3B—C3—H3C	109.5	C52—C58—H58A	109.5
Al1—C4—H4A	109.5	C52—C58—H58B	109.5
Al1—C4—H4B	109.5	H58A—C58—H58B	109.5
H4A—C4—H4B	109.5	C52—C58—H58C	109.5
Al1—C4—H4C	109.5	H58A—C58—H58C	109.5
H4A—C4—H4C	109.5	H58B—C58—H58C	109.5
H4B—C4—H4C	109.5	C60—C59—C64	120.0
C5—O2—La1	95.0 (2)	C60—C59—C65	124.4 (10)
C5—O3—La1^i^	152.7 (2)	C64—C59—C65	113.0 (12)
C5—O3—La1	94.8 (2)	C61—C60—C59	120.0
La1^i^—O3—La1	111.30 (9)	C61—C60—H60	120.0
O2—C5—O3	118.4 (3)	C59—C60—H60	120.0
O2—C5—C6	123.7 (3)	C60—C61—C62	120.0
O3—C5—C6	117.8 (3)	C60—C61—H61	120.0
O2—C5—La1	59.51 (19)	C62—C61—H61	120.0
O3—C5—La1	59.24 (19)	C63—C62—C61	120.0
C6—C5—La1	172.3 (2)	C63—C62—H62	120.0
C7—C6—C13	111.8 (3)	C61—C62—H62	120.0
C7—C6—C5	104.6 (3)	C62—C63—C64	120.0
C13—C6—C5	110.0 (3)	C62—C63—H63	120.0
C7—C6—C19	112.2 (3)	C64—C63—H63	120.0
C13—C6—C19	106.7 (3)	C63—C64—C59	120.0
C5—C6—C19	111.6 (3)	C63—C64—H64	120.0
C8—C7—C12	117.8 (4)	C59—C64—H64	120.0
C8—C7—C6	119.4 (3)	C59—C65—H65A	109.5
C12—C7—C6	122.2 (4)	C59—C65—H65B	109.5
C9—C8—C7	122.0 (4)	H65A—C65—H65B	109.5
C9—C8—H8	119.0	C59—C65—H65C	109.5
C7—C8—H8	119.0	H65A—C65—H65C	109.5
C10—C9—C8	119.5 (4)	H65B—C65—H65C	109.5
			
C4—Al1—O1—C1	65.1 (4)	O5—C25—C26—C39	−142.5 (3)
C3—Al1—O1—C1	−60.3 (4)	O4—C25—C26—C27	−77.6 (4)
C2—Al1—O1—C1	−177.7 (4)	O5—C25—C26—C27	98.5 (4)
La1—Al1—O1—C1	176.5 (4)	C33—C26—C27—C32	−108.6 (4)
C4—Al1—O1—La1	−111.4 (2)	C39—C26—C27—C32	13.0 (5)
C3—Al1—O1—La1	123.2 (2)	C25—C26—C27—C32	134.2 (4)
C2—Al1—O1—La1	5.8 (2)	C33—C26—C27—C28	68.4 (4)
La1—O2—C5—O3	−6.3 (4)	C39—C26—C27—C28	−170.0 (3)
La1—O2—C5—C6	171.8 (3)	C25—C26—C27—C28	−48.8 (4)
La1^i^—O3—C5—O2	169.6 (3)	C32—C27—C28—C29	−1.8 (6)
La1—O3—C5—O2	6.4 (4)	C26—C27—C28—C29	−179.0 (4)
La1^i^—O3—C5—C6	−8.6 (7)	C27—C28—C29—C30	1.3 (6)
La1—O3—C5—C6	−171.9 (3)	C28—C29—C30—C31	−0.1 (7)
La1^i^—O3—C5—La1	163.3 (5)	C29—C30—C31—C32	−0.5 (7)
O2—C5—C6—C7	170.6 (4)	C28—C27—C32—C31	1.2 (6)
O3—C5—C6—C7	−11.2 (4)	C26—C27—C32—C31	178.3 (4)
O2—C5—C6—C13	−69.2 (5)	C30—C31—C32—C27	−0.1 (7)
O3—C5—C6—C13	109.0 (4)	C39—C26—C33—C34	−112.0 (4)
O2—C5—C6—C19	49.1 (5)	C27—C26—C33—C34	9.8 (5)
O3—C5—C6—C19	−132.8 (3)	C25—C26—C33—C34	123.8 (4)
C13—C6—C7—C8	170.5 (3)	C39—C26—C33—C38	65.4 (4)
C5—C6—C7—C8	−70.4 (4)	C27—C26—C33—C38	−172.8 (3)
C19—C6—C7—C8	50.7 (5)	C25—C26—C33—C38	−58.7 (4)
C13—C6—C7—C12	−18.2 (5)	C38—C33—C34—C35	1.0 (6)
C5—C6—C7—C12	100.9 (4)	C26—C33—C34—C35	178.4 (4)
C19—C6—C7—C12	−138.0 (4)	C33—C34—C35—C36	−0.1 (7)
C12—C7—C8—C9	1.7 (6)	C34—C35—C36—C37	−0.9 (7)
C6—C7—C8—C9	173.3 (3)	C35—C36—C37—C38	1.0 (7)
C7—C8—C9—C10	0.0 (6)	C36—C37—C38—C33	−0.1 (6)
C8—C9—C10—C11	−1.0 (6)	C34—C33—C38—C37	−0.9 (6)
C9—C10—C11—C12	0.2 (6)	C26—C33—C38—C37	−178.5 (4)
C8—C7—C12—C11	−2.4 (6)	C33—C26—C39—C40	−169.7 (4)
C6—C7—C12—C11	−173.8 (3)	C27—C26—C39—C40	67.6 (4)
C10—C11—C12—C7	1.5 (6)	C25—C26—C39—C40	−47.9 (5)
C7—C6—C13—C14	−58.2 (4)	C33—C26—C39—C44	15.3 (5)
C5—C6—C13—C14	−173.9 (3)	C27—C26—C39—C44	−107.4 (4)
C19—C6—C13—C14	64.8 (4)	C25—C26—C39—C44	137.1 (4)
C7—C6—C13—C18	123.4 (4)	C44—C39—C40—C41	−3.2 (6)
C5—C6—C13—C18	7.6 (5)	C26—C39—C40—C41	−178.4 (4)
C19—C6—C13—C18	−113.7 (4)	C39—C40—C41—C42	2.6 (6)
C18—C13—C14—C15	−1.6 (6)	C40—C41—C42—C43	−0.6 (6)
C6—C13—C14—C15	179.8 (4)	C41—C42—C43—C44	−0.6 (7)
C13—C14—C15—C16	1.6 (7)	C42—C43—C44—C39	−0.2 (7)
C14—C15—C16—C17	−0.8 (7)	C40—C39—C44—C43	2.1 (6)
C15—C16—C17—C18	0.1 (7)	C26—C39—C44—C43	177.3 (4)
C16—C17—C18—C13	−0.2 (6)	C50—C45—C46—C47	1.0 (8)
C14—C13—C18—C17	1.0 (6)	C51—C45—C46—C47	−178.5 (5)
C6—C13—C18—C17	179.4 (3)	C45—C46—C47—C48	0.6 (9)
C7—C6—C19—C20	24.5 (5)	C46—C47—C48—C49	−2.0 (9)
C13—C6—C19—C20	−98.2 (4)	C47—C48—C49—C50	1.8 (9)
C5—C6—C19—C20	141.6 (4)	C46—C45—C50—C49	−1.2 (8)
C7—C6—C19—C24	−160.1 (3)	C51—C45—C50—C49	178.3 (5)
C13—C6—C19—C24	77.2 (4)	C48—C49—C50—C45	−0.2 (8)
C5—C6—C19—C24	−43.1 (5)	C57—C52—C53—C54	0.0
C24—C19—C20—C21	0.7 (6)	C58—C52—C53—C54	178.4 (16)
C6—C19—C20—C21	176.1 (4)	C52—C53—C54—C55	0.0
C19—C20—C21—C22	1.5 (7)	C53—C54—C55—C56	0.0
C20—C21—C22—C23	−2.7 (7)	C54—C55—C56—C57	0.0
C21—C22—C23—C24	1.6 (7)	C55—C56—C57—C52	0.0
C20—C19—C24—C23	−1.7 (6)	C53—C52—C57—C56	0.0
C6—C19—C24—C23	−177.2 (3)	C58—C52—C57—C56	−178.3 (18)
C22—C23—C24—C19	0.5 (6)	C64—C59—C60—C61	0.0
La1—O4—C25—O5	−7.1 (6)	C65—C59—C60—C61	−160.7 (16)
La1—O4—C25—C26	168.7 (2)	C59—C60—C61—C62	0.0
La1^i^—O5—C25—O4	6.7 (7)	C60—C61—C62—C63	0.0
La1^i^—O5—C25—C26	−169.3 (3)	C61—C62—C63—C64	0.0
O4—C25—C26—C33	163.5 (3)	C62—C63—C64—C59	0.0
O5—C25—C26—C33	−20.4 (5)	C60—C59—C64—C63	0.0
O4—C25—C26—C39	41.4 (5)	C65—C59—C64—C63	162.8 (13)

Symmetry code: (i) −*x*, −*y*+1, −*z*+1.

### Hydrogen-bond geometry (Å, º)

Cg1, Cg2, Cg3 and Cg4 are the centroids of the C33–C38, C39–C44, C52–C57 and C19–C24 rings, respectively.

**Table:**

*D*—H···*A*	*D*—H	H···*A*	*D*···*A*	*D*—H···*A*
C1—H1*C*······*Cg*1^i^	0.98	2.69	3.425 (6)	132
C17—H17······*Cg*2	0.95	2.71	3.485 (4)	139
C21—H21······*Cg*3^ii^	0.95	2.93	3.677 (8)	136
C29—H29······*Cg*4	0.95	2.62	3.415 (4)	142
C32—H32······*Cg*2	0.95	2.95	3.654 (5)	132
C44—H44······*Cg*1	0.95	2.88	3.592 (5)	132

Symmetry codes: (i) −*x*, −*y*+1, −*z*+1; (ii) −*x*+1, −*y*, −*z*+1.
